# Plasmons Enhancing Sub-Bandgap Photoconductivity in TiO_2_ Nanoparticles Film

**DOI:** 10.1021/acsomega.3c06932

**Published:** 2024-02-20

**Authors:** Mohammed A. Ibrahem, Emanuele Verrelli, Ali M. Adawi, Jean-Sebastien G. Bouillard, Mary O’Neill

**Affiliations:** † Laser Sciences and Technology Branch, Applied Sciences Department, University of Technology, Al-Sinaa Street, Baghdad 10066, Iraq; ‡ UNAM-Institute of Materials Science and Nanotechnology and National Nanotechnology Research Center, Bilkent University, Ankara 06800, Turkey; § Department of Physics and Mathematics, University of Hull, Cottingham Road, Kingston upon Hull HU6 7RX, United Kingdom; ∥ School of Science and Technology, Nottingham Trent University, Clifton Lane, Nottingham NG11 8NS, United Kingdom

## Abstract

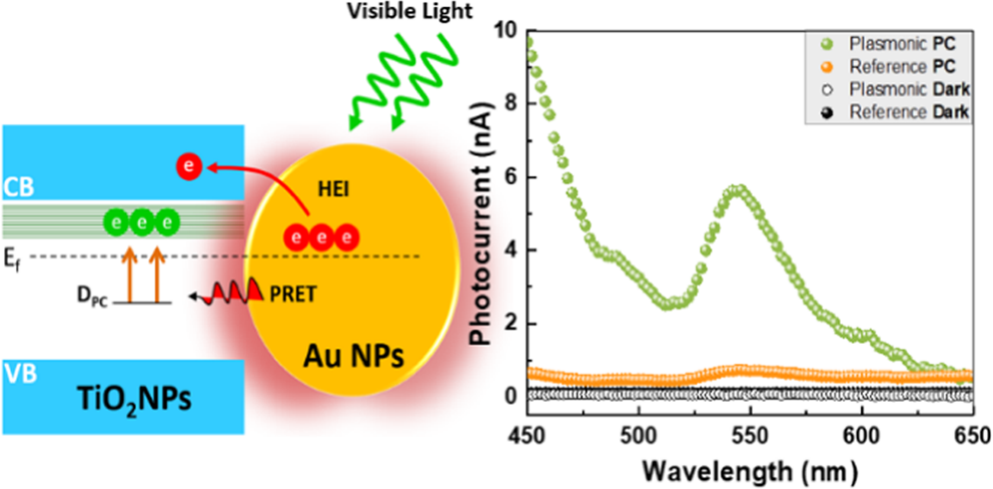

The coupling between sub-bandgap defect states and surface plasmon resonances in Au nanoparticles and its effects on the photoconductivity performance of TiO_2_ are investigated in both the ultraviolet (UV) and visible spectrum. Incorporating a 2 nm gold nanoparticle layer in the photodetector device architecture creates additional trapping pathways, resulting in a faster current decay under UV illumination and a significant enhancement in the visible photocurrent of TiO_2_, with an 8-fold enhancement of the defects-related photocurrent. We show that hot electron injection (HEI) and plasmonic resonance energy transfer (PRET) jointly contribute to the observed photoconductivity enhancement. In addition to shedding light on the below-band-edge photoconductivity of TiO_2_, our work provides insight into new methods to probe and examine the surface defects of metal oxide semiconductors using plasmonic resonances.

## 1 Introduction

Due to their potential for clean and sustainable technology, light harvesting materials and strategies have gained considerable attention in the last couple of years. TiO_2_ is extensively investigated due to its direct wide bandgap and tunable electrical and optical properties that originate from its intragap energy levels related to inherited surface defects and oxygen vacancies.^1,2^ Such unique properties enable TiO_2_ to be highly desirable in light harvesting applications such as photocatalysis,^3^ photovoltaics,^4^ photodetection,^5^ and photoelectrochemical sensing.^6^ Due to its hypotoxicity and abundance, developing efficient photoconductivity of TiO_2_ in the visible band will significantly impact the environment and device production cost.^7^ Several attempts to extend the optical absorption of TiO_2_ to the visible spectral region by doping with different materials have been reported.^3,8−10^ In parallel, reducing the bandgap of TiO_2_ by defects engineering has also been considered by deliberately introducing Ti^3+^, lattice disorder defects, and oxygen defects within the crystalline structure of TiO_2_.^11−13^ However, these attempts often lack long-term stability and repeatability.^14^

As an attractive alternative, the strong field enhancement and confinement of localized surface plasmon resonances (LSPR) represent an efficient strategy to stimulate visible photoresponses in many metal oxide semiconductors. Possible mechanisms are plasmonic energy conversion by hot electron injection (HEI) to a nearby semiconductor^15,16^ and near-field coupling and energy transfer, sometimes called plasmonic resonance energy transfer (PRET).^17^ Near-field coupling, or PRET, requires spectral overlap between the semiconductor absorption and the LSPR, whereas HEI relies on the injection of hot charge carriers from the plasmon decay through the Schottky barrier.^6,18,19^

TiO_2_-based hybrid systems have been widely studied to enhance and extend the optoelectronic response of TiO_2_ into the visible and near-IR, enabling a wide range of applications from photocatalysis to sensing and photodetectors.^6,8,16,20−28^ Shu et al.^6^ investigated the coupling interaction of surface defects engineered in TiO_2_ nanobars with the plasmonic field of gold nanoparticles (Au NPs) in the UV and visible for photoelectrochemical biosensing applications. Their results show a 25% reduction in UV response combined with a 6- to 7-fold increase in the visible photoconductivity, in the presence of Au NPs. Chen et al.^20^ reported visible surface defect photocurrent enhancement in TiO_2_:Au nanocomposite film due to electron–hole pairs generated via PRET and HEI. Tan et al.^29^ extended this to the near-IR using a TiO_2_–Au bilayer. Naldoni et al.^30^ observed a similar behavior by introducing oxygen defect states via doping of the TiO_2_ and highlighted the importance of both the interband transitions and the plasmonic resonance in TiO_2_:Au systems.

In this work, we report on unusually spectrally narrow defects photocurrent in the visible region in a vertical metal–semiconductor–metal (MSM) device configuration, with a full width at half-maximum (fwhm) under 40 nm obtained for illumination at a wavelength of ∼545 nm. Unlike conventional charge collection devices, our device structure shows a defined defect-based visible photocurrent under zero bias, with the visible photocurrent showing an 8-fold enhancement when decorated with thermally evaporated Au nanoparticles. This significant enhancement in photocurrent is attributed to the combined effect of HEI and near-field/PRET processes stimulated by the plasmonic resonance of the Au nanostructures interfaced with TiO_2_. Our findings can assist the study of localized surface defects in wide-bandgap semiconductors; they also represent a strategy to enhance the selective photocatalytic reaction of TiO_2_ and develop an advanced photoelectric material for biosensing applications.

## 2 Results and Discussion

**Figure 1:**
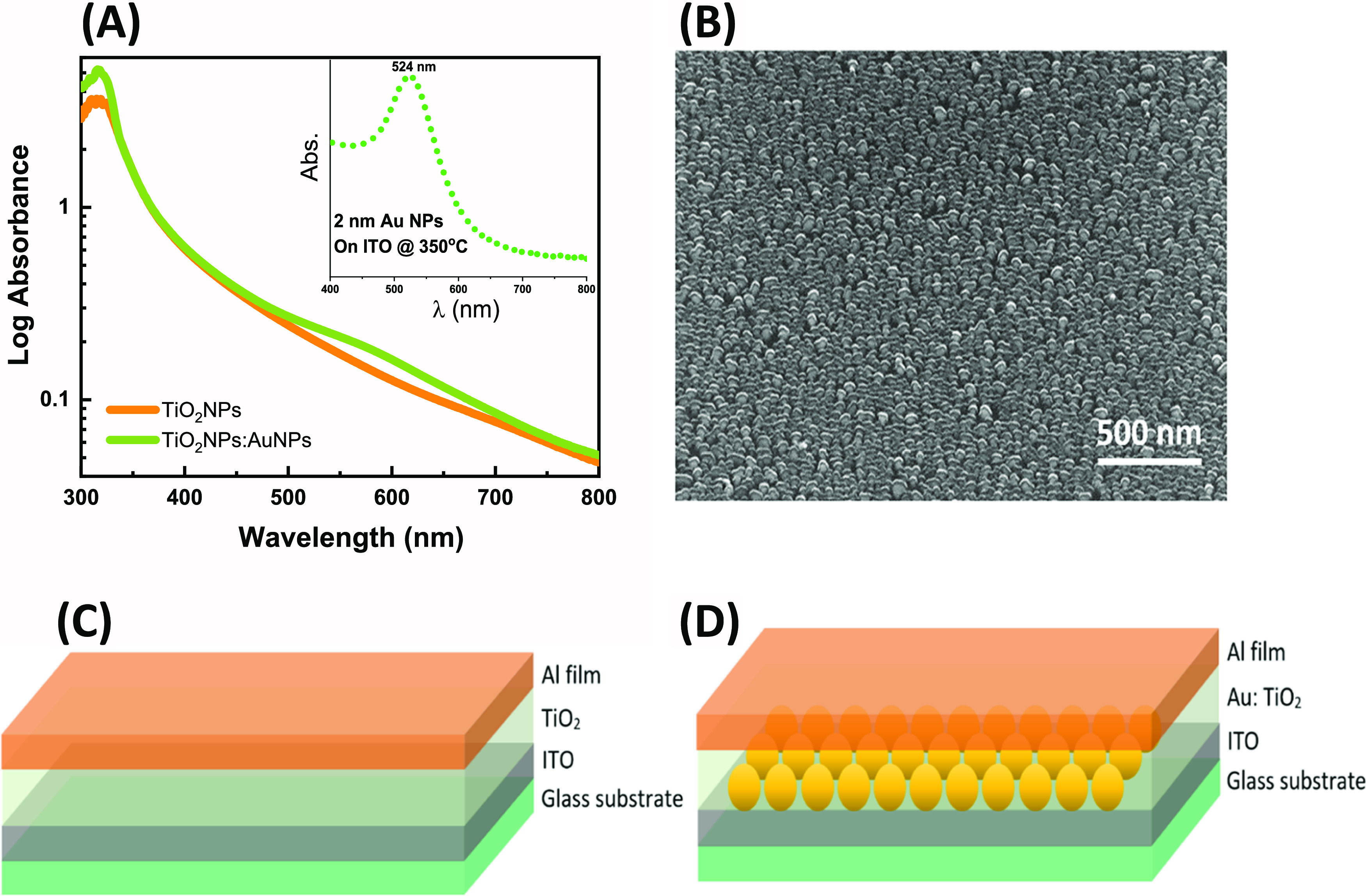
(A) Semilog plot of the absorption spectra of TiO_2_ NPs films with and without Au NPs. The inset shows the absorption spectrum of ITO\Au NPs after annealing for 1 h at 350 °C in air (shown in (B)). (B) SEM image of Au NPs film on ITO substrate after annealing at 350 °C for 1 h in air. (C, D) Structures and configuration of the metal–semiconductor–metal devices utilized in this work, the reference device with no Au NPs and the plasmonic device with Au NPs layer, respectively.

Figure 1A shows the optical absorption spectra of TiO_2_ nanoparticle (TiO_2_ NP) films with and without the Au NPs layer. The absorption peak around a wavelength of 322 nm (3.8 eV) corresponds to the fundamental band-to-band electronic transition. This absorption peak occurs at a higher energy than the expected transition energy of TiO_2_ (3.2 eV) and can be attributed to the quantum confinement effect of the TiO_2_ nanoparticles.^31^ The plot also shows a long absorption tail that decays into the visible spectrum region, attributed to surface defects.^20,32^ The visible absorption is further increased after the incorporation of Au nanoparticles due to the plasmonic resonance effect. This plasmonic absorption peak is red-shifted compared to the Au nanoparticles absorption on ITO in air (before the deposition of TiO_2_ NPs film shown in the inset of Figure 1A) due to the higher refractive index of the TiO_2_ nanoparticles film now surrounding the Au NPs.

**Figure 2:**
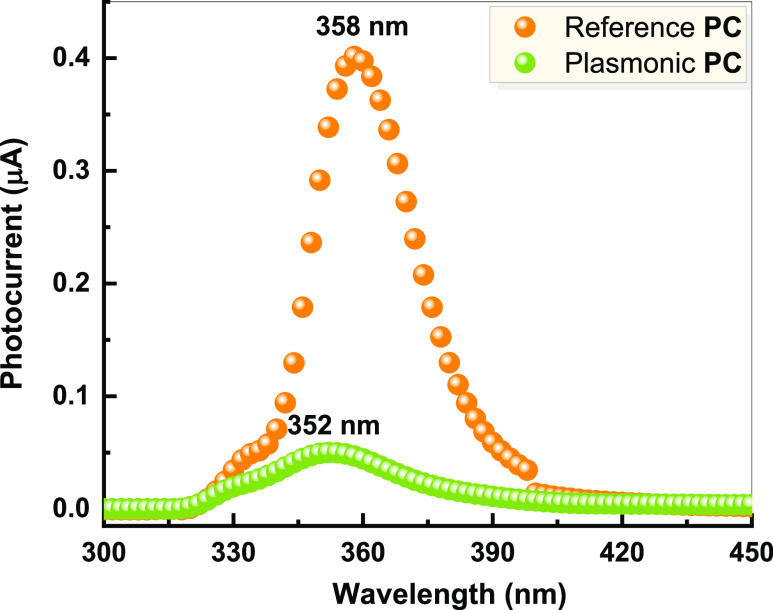
Photocurrent spectra of the reference and the plasmonic devices at 0 V bias showing the photocurrent scanning range from 300–450 nm.

The photoconductivity of both the reference and the plasmonic devices shows the characteristic UV response of the TiO_2_ nanoparticle films, arising from the direct band-to-band electron transitions and the visible band arising from defect states.^5,33^Figure 2 shows the photoconductivity spectrum of each device at 0 V bias over the spectral range of 300 to 450 nm. Both devices show photoconductivity in the UV, reaching a photocurrent of 0.4 and 0.054 μA for the reference and plasmonic devices, respectively. The drop in UV photocurrent in the presence of gold nanoparticles is associated with additional charge trapping at the metal–semiconductor interface and electron transfer from the TiO_2_ to the Au NPs.^20,34^ In parallel, the addition of the Au NPs causes band bending and charge trapping at the TiO_2_–Au interface,^35,36^ resulting in the observed blue shift in the UV photocurrent from 358 to 352 nm, with corresponding increased fwhm from 27.4 to 36.3 nm (Figure 2).

**Figure 3:**
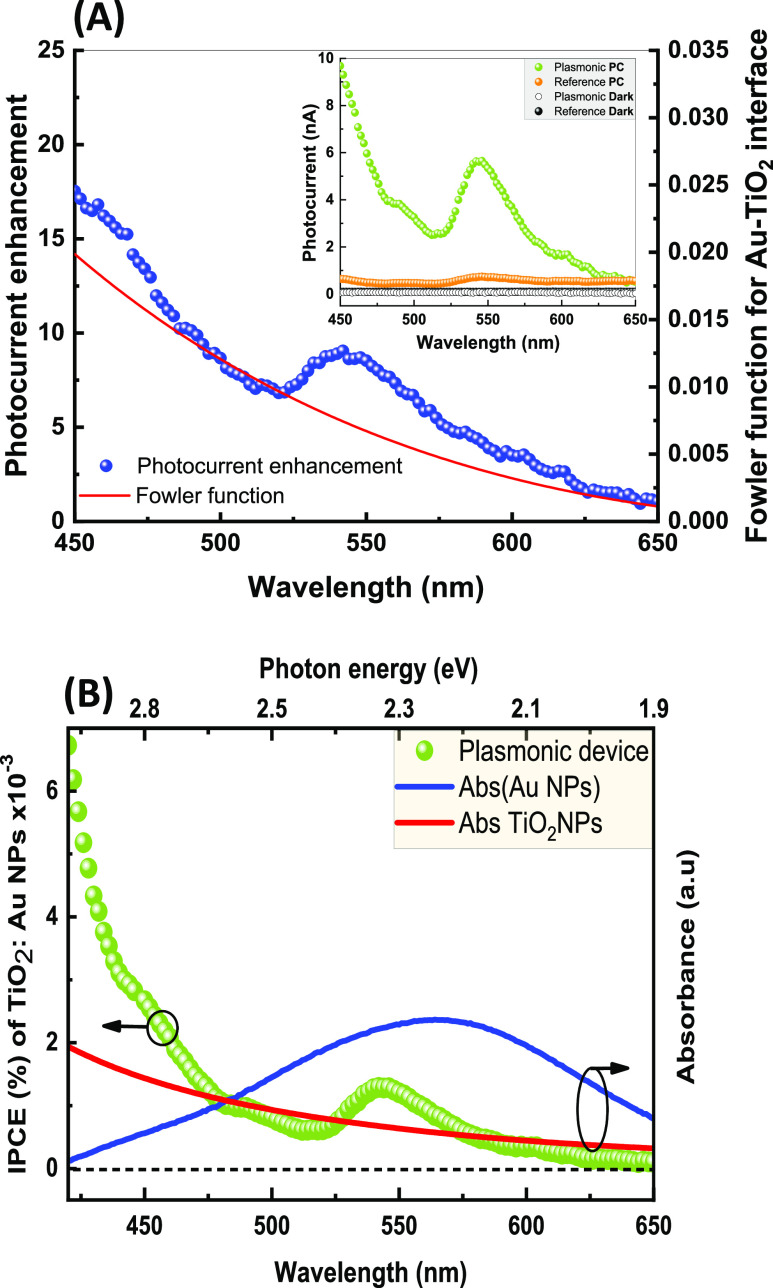
(A) Photocurrent enhancement spectrum (normalized to the reference device) of the plasmonic device at 0 V bias with the Fowler function for a gold–TiO_2_ interface. The inset shows the photocurrent spectra of the reference and the plasmonic devices at 0 V bias (normalized to the light spectrum intensity), along with the dark current for each device. (B) IPCE of the TiO_2_NPs-incorporated Au NPs showing the spectral absorption overlap between defects-related absorption spectrum (red curve) and the plasmonic absorption of Au NPs (blue curve) acquired by subtracting the absorption of TiO_2_ NPs film from the absorption of TiO_2_ NPs + Au NPs film shown in Figure 1A.

In addition to the UV response observed in Figure 2, a clear photocurrent enhancement in the visible range (450–650 nm) can be observed (Figure 3A). This photocurrent increases at energies well below the TiO_2_ bandgap and can be linked to HEI, where the plasmon decay results in hot electrons with sufficient energy to overcome the Schottky barrier. This is illustrated by the calculated Fowler function for a gold–TiO_2_ interface^37−42^ (Figure 3A).

Additionally, both devices show a pronounced photocurrent peak around 545 nm (inset of Figure 3A), corresponding to the transition between the surface defect and the conduction band in TiO_2_,^43^ with a photoconductivity 8 times larger in the plasmonic device than the reference device. This can be attributed to the dipole interaction between surface plasmons and sub-bandgap energy states of surface defects through the PRET process, where the strong plasmonic near-field enhancement of Au NPs leads to an efficient transition between the surface defect and the conduction band in TiO_2_. Another photocurrent peak evolved at higher photon energy, below the plasmonic resonance of Au NPs (458 nm), and corresponds to light absorption in gold due to electronic transitions from the d-bands to the sp-bands.^44,45^ The photocurrent enhancement at this shorter wavelength is mainly related to the optical absorption of TiO_2_ NPs and scattering by the Au NPs. This is further supported by the spectral overlap between the incident photon-to-electron conversion efficiency (IPCE) of the plasmonic device and the absorption spectrum of the gold nanoparticles inside the device, normalized to the reference device to remove the TiO_2_ component shown in Figure 3B.

**Figure 4:**
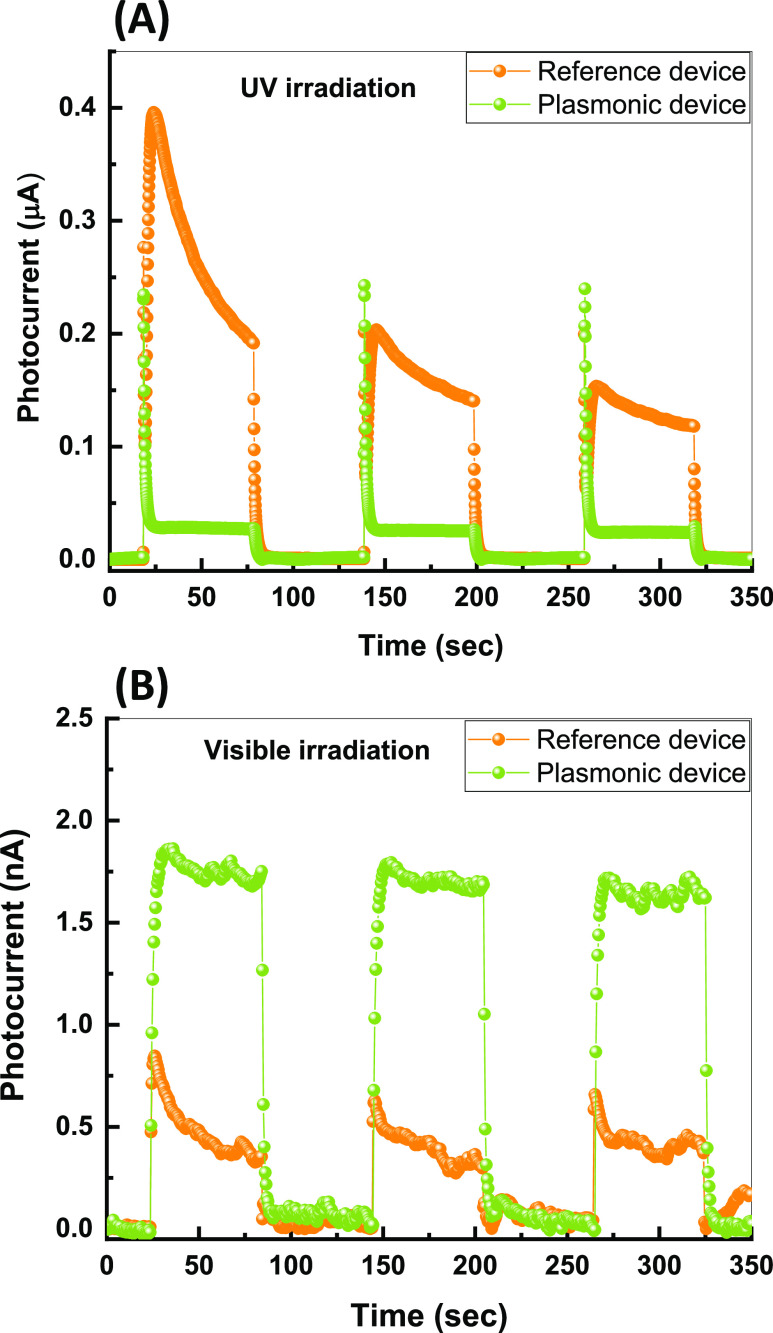
Photocurrent of the reference and the plasmonic devices as a function of time at 0 V bias in response to a 1 min incident light pulse at (A) UV and (B) visible.

To probe the photocurrent dynamics, the temporal response of the reference and the plasmonic devices were recorded at 0 V under both UV (355 ± 5 nm) and visible (545 ± 5 nm) irradiation, in 1 min on/off cycles as shown in Figure 4A,B. Both devices show a fast rise and decay in photocurrent when UV irradiation is switched on and off, with an order of magnitude higher photocurrent from the reference device than the plasmonic one (Figure 4A). The rise and decay time constants were measured to be τ_r_ = 5.9 s and τ_d_ = 6 s, respectively, for the reference device, and τ_r_ = 4.4 s and τ_d_ = 3.8 s for the plasmonic device. Table 1 provides a comparison of the UV temporal responses of TiO_2_-based photodetector devices and our work.

**Table 1:** Comparison of the Temporal Responses of Our Work and Those of Previous Reports

		UV photoresponse			visible/NIR photoresponse			
photodetector	bias (V)	**λ** (nm)	**τ**_**r**_ (*s*)	**τ**_**d**_ (*s*)	**λ** (nm)	**τ**_**r**_ (*s*)	**τ**_**d**_ (*s*)	refs
TiO_2_ film	5	365	0.017	0.019				(46)
TiO_2_ NTs/Ag NPs	1	365	0.43	0.70				(47)
TiO_2_ NRs/Ag NPs	1	365	3.90	5.70				(47)
TiO_2_/Ag porous films	0	450	112 μs	24 μs	660			(48)
TiO_2_ NWs/Ag NPs	3	350	0.56	0.13	white light	0.24	0.14	(49)
TiO_2–*x*_/Au NPs		365			450,520, 585, 630			(6)
TiO_2_/spiro-MeOTAD	0				410	0.12	0.06	(50)
Au/TiO_2_/ NR/Au	–4	350			400,470,570			(51)
ZnO NPs	20	363	>0.5		545	6.5	τ_d1_ = 2.5	(1)
							τ_d2_ = 18.8	
					596	14.5	τ_d1_ = 6.6	
							τ_d2_ = 25.6	
ZnO NPs/Au	0.01	363			850	τ_r1_ = 9.5	τ_d1_ = 15.5	(16)
						τ_r2_ = 73.5	τ_d2_ = 81.7	
ZnO NWs/Au NRs					650	τ_r1_ = 6.3	τ_d1_ = 16.5	(52)
						τ_r2_ = 0.238	τ_d2_ = 0.69	
					850			
MoS_2_/Au					532	τ_r1_ = 28.5	232	(53)
						τ_r2_ = 494.3		
					1070	τ_r1_ = 44.5	216.5	
						τ_r2_ = 404.7		
this work	0	355	4.4	3.8	545	5.6	4.5	

The reference device shows a slow exponential-like decay of the photocurrent even when illumination is maintained (Figure 4A). This reduction in photocurrent during illumination is attributed to charge trapping of the charge carriers on the surface of TiO_2_ after excitation.^62,63^ In addition, the peak photocurrent in the reference device decreases with each illumination cycle, highlighting long-lived processes, potentially arising from charge accumulation in trap states, impeding the charge carrier flow in the device. On the other hand, the plasmonic device shows a spike-like photocurrent signal followed by a significantly lower but stable photocurrent. The incorporation of Au NPs in the plasmonic device creates additional effective trapping pathways, such as interfacial sites at the TiO_2_–Au,^35^ leading to a faster decay in the UV photocurrent, overcoming the long-lived trap states, resulting in the observed photocurrent (Figure 4A).

Figure 4B shows the temporal responses of the reference and plasmonic devices upon visible illumination. The incorporation of Au NPs has significantly enhanced the sub-bandgap photoconductivity compared to the reference device (with no Au NPs) due to the effect of the plasmonic resonance excitation of electrons in the Au NPs. The corresponding time constants were measured as τ_r_ = 2.5 and τ_d_ = 5 s for the reference device, and τ_r_ = 5.6 and τ_d_ = 4.5 s for the plasmonic device. This temporal response of the plasmonic device is comparable to recently reported time responses for TiO_2_/Au NPs^64,65^ and slower than the self-powered heterojunction device based on Cul/TiO_2_^66^ and could be used for UV switching applications with frequencies of a few hertz.

The principal mechanism by which photogenerated charge carriers move in our plasmonic device differs from that of conventional charge collection devices working with external bias. In such devices, electrons and holes move in opposite directions, following the electric field provided by the external source. Furthermore, the photoresponse time will highly depend on environmental conditions, such as the oxygen adsorption/desorption process, which often leads to long photoresponse times.^1^ On the contrary, in our plasmonic device, charge carriers are separated and driven in opposite directions by the built-in photovoltaic field stimulated from the Schottky junction established at the interface between Au NPs and TiO_2_ nanoparticles film. This enables the plasmonic device to have a fast photoresponse without needing an external field source.^50^

**Figure 5:**
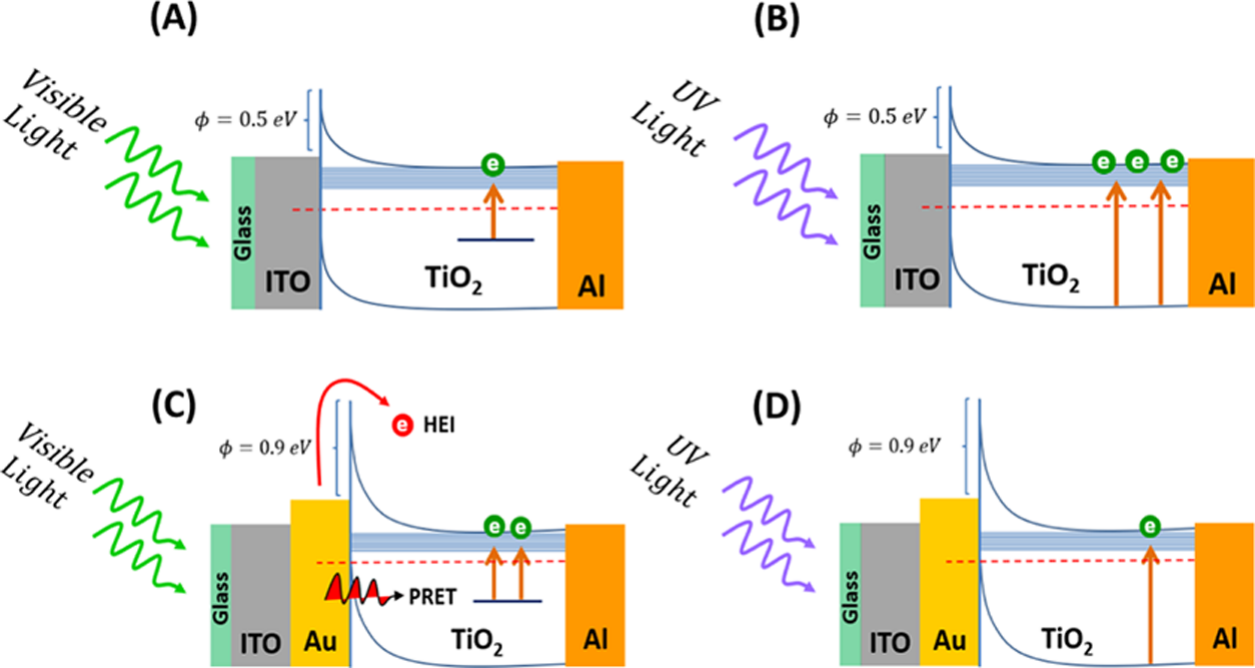
Schematic energy band diagrams illustrating the photoconductivity mechanisms for the reference device upon light irradiation in the (A) visible and (B) UV regions, and the plasmonic device upon light irradiation in the (C) visible and (D) UV regions. Hot electron injection (HEI) and plasmonic resonance energy transfer (PRET) are responsible for the visible photocurrent enhancement after the incorporation of Au nanoparticles in the plasmonic device.

A physical picture showing the working mechanism of the devices with and without the incorporation of Au NPs upon UV and visible light irradiation is schematically illustrated in Figure 5. In the reference device, UV photocurrent corresponds to the standard transition from the valence band to the conduction band, whereas the visible photocurrent arises from the excitation of electrons from sub-bandgap energy states into the conduction band of the TiO_2_ (Figure 5A,B). The Schottky junction between the ITO (work function: 4.7 eV) and the TiO_2_ NPs (conduction band edge: 4.2 eV)^70^ with a junction height estimated to be 0.5 eV is the main driving force of the photo charge carriers’ dissociation, which powers the device without needing an external field.

However, in the plasmonic device, the Schottky junction between the Au NPs (work function: 5.1 eV) and the TiO_2_ NPs with an overall junction height of about 0.9 eV (according to band alignment) creates the internal electric field, forcing the photogenerated electrons and holes to move in opposite directions.^71^ The smaller Schottky height in the reference device explains the higher UV photocurrent than that in the plasmonic device. The existence of Au NPs can also trap the photo charge carriers by acting as a charge sink due to a work function difference, which will further reduce the UV photocurrent in the plasmonic device.^30,35^ Due to the relatively small difference in work function between the TiO_2_ and the aluminum (Al) back electrode (4.3 eV),^57,58^ the junction can be treated as an ohmic contact.

In the case of visible light irradiation, on the other hand, electrons trapped at the surface defects will be excited to the conduction band leading to sub-bandgap photoactivity. The observed 8-fold enhancement in visible photoconductivity in the plasmonic device is attributed to the coupling between the sub-bandgap defect states and the surface plasmons of Au nanoparticles. Two plasmonic mechanisms are jointly responsible for the sub-bandgap photocurrent enhancement in TiO_2_ in the plasmonic device: hot electrons injection (HEI) and plasmonic resonance energy transfer (PRET), illustrated in Figure 5C. These plasmonic mechanisms mainly enhance the photoconductivity in semiconductor devices,^6,59,60^ despite the higher Schottky barrier; however, their active engagement with the sub-bandgap defect states is not yet considered widely in the literature.

## 3 Conclusions

In summary, a spectrally narrow photoresponse in the visible region was observed from a 20 nm TiO_2_ NPs (P25) layer in a vertical configuration metal–semiconductor–metal device at 0 applied voltage. This significant sub-bandgap photocurrent is attributed to the inherited surface defects and liberation of trapped electrons to the conduction band. Defect electron transition is found to be enhanced significantly after the incorporation of Au nanoparticles. HEI and PRET are jointly responsible for this enhancement due to the direct contact between Au nanoparticles and TiO_2_ nanoparticles film and the excellent overlap between the plasmonic resonance of the Au NPs and the absorption spectrum of the surface defects of the TiO_2_ nanoparticles film. This study outlines the active interaction between surface defects and surface plasmons, which could be highly beneficial in studying surface defects in semiconductors. A physical model explaining charge injection and separation based on HEI and PRET is proposed. Our findings could be of interest in imaging applications where a solution-processed TiO_2_ photodetector, with different photoresponses, UV and visible, is possible. It also benefits those who are investigating selective photocatalytic reactions based on TiO_2_.

## 4 Experimental Section

Two different devices with vertical configuration structures, a reference device (ITO/TiO_2_NPs/Al) and a plasmonic device (ITO/Au/TiO_2_NPs/Al), were considered (Figure 1C,D). Devices were fabricated on a float glass substrate with prepatterned interdigitated indium tin oxide (ITO) electrodes (20 Ω/square) with overall channel dimensions (device area) of 3 mm × 1.5 mm, (Ossila Ltd.). ITO substrates were thoroughly cleaned using an ultrasonic bath in three consecutive solutions, acetone, ethanol, and distilled water, and then dried with N_2_. The substrates were then treated with UV/ozone for 3 min to remove any organic contaminants and impurities remaining on the surface and improve surface wettability.

The plasmonic device was fabricated by thermally evaporating Au directly on the ITO substrate with a deposition rate of 0.1 Å/s at 5 × 10^–7^ mbar using an HHV Auto 500 multifunctional automated evaporator, resulting in a nominal thickness of 2 nm. The sample was then annealed in air at 350 °C for 1 h, leading to the dewetting of the Au layer, resulting in a dense layer of Au nanoparticles with 45 ± 5 nm diameter distributed uniformly over the substrate (Figure 1B).

For both the plasmonic and reference devices, TiO_2_ nanoparticles suspension of concentration 10% by weight in deionized water was prepared using commercial TiO_2_ NPs powder (P25) with a particle diameter of 20 nm bought from Sigma-Aldrich, containing a mix of rutile and anatase phases. Before deposition, the solution was filtered with a 0.45 μm poly(tetrafluoroethylene) (PTFE) syringe filter to improve film quality.

The filtered suspension of TiO_2_ NPs is then spin-cast on top of Au NPs at a speed of 2000 rpm for 30 s. These deposition conditions were optimized to obtain TiO_2_ nanoparticle films of 200 nm thickness (±5 nm) measured using a Dektak profilometer from Bruker. The overall hybrid structure was baked in air at 150 °C for 10 min before a 150 nm aluminum (Al) film was thermally evaporated to form the device back electrode. A reference device was fabricated following the same fabrication procedure without incorporating the Au NPs layer. A thickness of 200 nm was used for the TiO_2_ nanoparticle film in order to avoid short-circuiting the device when depositing the top electrode (Al) through this porous layer.

Optical absorption was measured before evaporating the back Al electrode using a Thermo Scientific (EVOLUTION 220) spectrophotometer over the 300 to 900 nm spectral range. *I*–*V* and photocurrent measurements were recorded using a xenon lamp with a 5 nm bandwidth monochromator and two electrical needle probes interfaced to a Keithley 2400 source meter using BenWin+ software to record the photocurrent as a function of wavelength and applied voltage. The light intensity was monitored using a digital compact power and energy meter console with calibrated photodiode sensors from Thorlabs (PM100D). Devices were irradiated at a normal incident angle through the glass/ITO side, with the light beam covering the whole device’s active area. All of the measurements were done at room temperature in the air.

## Supporting Information Available

*I*–*V* curves of the photodetector devices under dark and visible irradiation, optical images of the photodetector device and Au NPs films, and explanation of Fowler function (PDF)

## References

[ref1] Ibrahem M. A.; Verrelli E.; Lai K. T.; Kyriakou G.; Lee A. F.; Isaacs M. A.; Cheng F.; O’Neill M. Dual Wavelength (Ultraviolet and Green) Photodetectors Using Solution Processed Zinc Oxide Nanoparticles. ACS Appl. Mater. Interfaces 2017, 9 (42), 36971–36979. 10.1021/acsami.7b08092. 28950063

[ref2] Banerjee S.; Pillai S. C.; Falaras P.; O’Shea K. E.; Byrne J. A.; Dionysiou D. D. New Insights into the Mechanism of Visible Light Photocatalysis. J. Phys. Chem. Lett. 2014, 5 (15), 2543–2554. 10.1021/jz501030x. 26277942

[ref3] Suwondo K. P.; Aprilita N. H.; Wahyuni E. T. Enhancement of TiO2 Photocatalytic Activity under Visible Light by Doping with Cu from Electroplating Wastewater. React. Kinet., Mech. Catal. 2022, 135 (1), 479–497. 10.1007/s11144-021-02134-1.

[ref4] Ding Y.; Ding B.; Kanda H.; Usiobo O. J.; Gallet T.; Yang Z.; Liu Y.; Huang H.; Sheng J.; Liu C.; et al. Single-Crystalline TiO2 Nanoparticles for Stable and Efficient Perovskite Modules. Nat. Nanotechnol. 2022, 17 (6), 598–605. 10.1038/s41565-022-01108-1. 35449409

[ref5] Nunes D.; Fortunato E.; Martins R. Flexible Nanostructured TiO2-Based Gas and UV Sensors: A Review. Discovery Mater. 2022, 2 (1), 2 10.1007/s43939-022-00023-5.

[ref6] Shu J.; Qiu Z.; Lv S.; Zhang K.; Tang D. Plasmonic Enhancement Coupling with Defect-Engineered TiO 2– X: A Mode for Sensitive Photoelectrochemical Biosensing. Anal. Chem. 2018, 90 (4), 2425–2429. 10.1021/acs.analchem.7b05296. 29397702

[ref7] Dharma H. N. C.; Jaafar J.; Widiastuti N.; Matsuyama H.; Rajabsadeh S.; Othman M. H. D.; Rahman M. A.; Jafri N. N. M.; Suhaimin N. S.; Nasir A. M.; Alias N. H. A Review of Titanium Dioxide (TiO2)-Based Photocatalyst for Oilfield-Produced Water Treatment. Membranes 2022, 12 (3), 345 10.3390/membranes12030345. 35323821 PMC8950424

[ref8] Xie Y.; Wei L.; Li Q.; Chen Y.; Liu H.; Yan S.; Jiao J.; Liu G.; Mei L. A High Performance Quasi-Solid-State Self-Powered UV Photodetector Based on TiO2 Nanorod Arrays. Nanoscale 2014, 6 (15), 9116–9121. 10.1039/C4NR01665C. 24974943

[ref9] Imran M.; Saeed Z.; Pervaiz M.; Mehmood K.; Ejaz R.; Younas U.; Nadeem H. A.; Hussain S. Enhanced Visible Light Photocatalytic Activity of TiO2 Co-Doped with Fe, Co, and S for Degradation of Cango Red. Spectrochim. Acta, Part A 2021, 255, 119644 10.1016/j.saa.2021.119644. 33812235

[ref10] Sirivallop A.; Areerob T.; Chiarakorn S. Enhanced Visible Light Photocatalytic Activity of N and Ag Doped and Co-Doped TiO2 Synthesized by Using an In-Situ Solvothermal Method for Gas Phase Ammonia Removal. Catalysts 2020, 10 (2), 251 10.3390/catal10020251.

[ref11] Xu Y.; Wu S.; Wan P.; Sun J.; Hood Z. D. Introducing Ti 3+ Defects Based on Lattice Distortion for Enhanced Visible Light Photoreactivity in TiO 2 Microspheres. RSC Adv. 2017, 7 (52), 32461–32467. 10.1039/C7RA04885H.

[ref12] Pan L.; Wang S.; Xie J.; Wang L.; Zhang X.; Zou J.-J. Constructing TiO2 P-n Homojunction for Photoelectrochemical and Photocatalytic Hydrogen Generation. Nano Energy 2016, 28, 296–303. 10.1016/j.nanoen.2016.08.054.

[ref13] Chen X.; Liu L.; Huang F. Black Titanium Dioxide (TiO 2) Nanomaterials. Chem. Soc. Rev. 2015, 44 (7), 1861–1885. 10.1039/C4CS00330F. 25590565

[ref14] Wang B.; Kerr L. L. Stability of CdS-Coated TiO2 Solar Cells. J. Solid State Electrochem. 2012, 16 (3), 1091–1097. 10.1007/s10008-011-1496-3.

[ref15] Ng C.; Cadusch J. J.; Dligatch S.; Roberts A.; Davis T. J.; Mulvaney P.; Gómez D. E. Hot Carrier Extraction with Plasmonic Broadband Absorbers. ACS Nano 2016, 10 (4), 4704–4711. 10.1021/acsnano.6b01108. 26982625

[ref16] Ibrahem M. A.; Verrelli E.; Cheng F.; Adawi A. M.; Bouillard J.-S. G.; O’Neill M. Persistent Near-Infrared Photoconductivity of ZnO Nanoparticles Based on Plasmonic Hot Charge Carriers. J. Appl. Phys. 2022, 131 (10), 103103 10.1063/5.0079006.

[ref17] Cushing S. K.; Li J.; Meng F.; Senty T. R.; Suri S.; Zhi M.; Li M.; Bristow A. D.; Wu N. Photocatalytic Activity Enhanced by Plasmonic Resonant Energy Transfer from Metal to Semiconductor. J. Am. Chem. Soc. 2012, 134 (36), 15033–15041. 10.1021/ja305603t. 22891916

[ref18] Naldoni A.; Allieta M.; Santangelo S.; Marelli M.; Fabbri F.; Cappelli S.; Bianchi C. L.; Psaro R.; Dal Santo V. Effect of Nature and Location of Defects on Bandgap Narrowing in Black TiO 2 Nanoparticles. J. Am. Chem. Soc. 2012, 134 (18), 7600–7603. 10.1021/ja3012676. 22519668

[ref19] Pan X.; Xu Y.-J. Defect-Mediated Growth of Noble-Metal (Ag, Pt, and Pd) Nanoparticles on TiO 2 with Oxygen Vacancies for Photocatalytic Redox Reactions under Visible Light. J. Phys. Chem. C 2013, 117 (35), 17996–18005. 10.1021/jp4064802.

[ref20] Chen W.; Lu Y.; Dong W.; Chen Z.; Shen M. Plasmon Mediated Visible Light Photocurrent and Photoelectrochemical Hydrogen Generation Using Au Nanoparticles/TiO2 Electrode. Mater. Res. Bull. 2014, 50, 31–35. 10.1016/j.materresbull.2013.10.017.

[ref21] Li X.; Gao C.; Duan H.; Lu B.; Pan X.; Xie E. Nanocrystalline TiO2 Film Based Photoelectrochemical Cell as Self-Powered UV-Photodetector. Nano Energy 2012, 1 (4), 640–645. 10.1016/j.nanoen.2012.05.003.

[ref22] Wang Z.; Ran S.; Liu B.; Chen D.; Shen G. Multilayer TiO2 Nanorod Cloth/Nanorod Array Electrode for Dye-Sensitized Solar Cells and Self-Powered UV Detectors. Nanoscale 2012, 4 (11), 3350–3358. 10.1039/c2nr30440f. 22549639

[ref23] Lee W.-J.; Hon M.-H. An Ultraviolet Photo-Detector Based on TiO 2 /Water Solid-Liquid Heterojunction. Appl. Phys. Lett. 2011, 99 (25), 251102 10.1063/1.3671076.

[ref24] Cao C.; Hu C.; Wang X.; Wang S.; Tian Y.; Zhang H. UV Sensor Based on TiO2 Nanorod Arrays on FTO Thin Film. Sensors Actuators, B 2011, 156 (1), 114–119. 10.1016/j.snb.2011.03.080.

[ref25] Chen J.; Hu B.; Chen C.; Lv X.; San H.; Hofmann W. In A High-Performance Self-Powered UV Photodetector Based on Self-Doping TIO 2 Nanotube Arrays, 2019 20th International Conference on Solid-State Sensors, Actuators and Microsystems & Eurosensors XXXIII (TRANSDUCERS & EUROSENSORS XXXIII); IEEE, 2019; pp 1329–1332.

[ref26] Gao Y.; Xu J.; Shi S.; Dong H.; Cheng Y.; Wei C.; Zhang X.; Yin S.; Li L. TiO 2 Nanorod Arrays Based Self-Powered UV Photodetector: Heterojunction with NiO Nanoflakes and Enhanced UV Photoresponse. ACS Appl. Mater. Interfaces 2018, 10 (13), 11269–11279. 10.1021/acsami.7b18815. 29558104

[ref27] Ferhati H.; Djeffal F.; Martin N. Highly Improved Responsivity of Self-Powered UV–Visible Photodetector Based on TiO2/Ag/TiO2Multilayer Deposited by GLAD Technique: Effects of Oriented Columns and Nano-Sculptured Surface. Appl. Surf. Sci. 2020, 529, 147069 10.1016/j.apsusc.2020.147069.

[ref28] Chen J.; Xu J.; Shi S.; Cao R.; Liu D.; Bu Y.; Yang P.; Xu J.; Zhang X.; Li L. Novel Self-Powered Photodetector with Binary Photoswitching Based on SnS x /TiO 2 Heterojunctions. ACS Appl. Mater. Interfaces 2020, 12 (20), 23145–23154. 10.1021/acsami.0c05247. 32338868

[ref29] Tan F.; Li T.; Wang N.; Lai S. K.; Tsoi C. C.; Yu W.; Zhang X. Rough Gold Films as Broadband Absorbers for Plasmonic Enhancement of TiO2 Photocurrent over 400–800 Nm. Sci. Rep. 2016, 6, 33049 10.1038/srep33049. 27608836 PMC5016800

[ref30] Naldoni A.; Fabbri F.; Altomare M.; Marelli M.; Psaro R.; Selli E.; Salviati G.; Dal Santo V. The Critical Role of Intragap States in the Energy Transfer from Gold Nanoparticles to TiO 2. Phys. Chem. Chem. Phys. 2015, 17 (7), 4864–4869. 10.1039/C4CP05775A. 25607570

[ref31] Xue X.; Ji W.; Mao Z.; Mao H.; Wang Y.; Wang X.; Ruan W.; Zhao B.; Lombardi J. R. Raman Investigation of Nanosized TiO 2: Effect of Crystallite Size and Quantum Confinement. J. Phys. Chem. C 2012, 116 (15), 8792–8797. 10.1021/jp2122196.

[ref32] Gorzkowska–Sobas A.; Kusior E.; Radecka M.; Zakrzewska K. Visible Photocurrent Response of TiO2 Anode. Surf. Sci. 2006, 600 (18), 3964–3970. 10.1016/j.susc.2006.01.108.

[ref33] Chen Z.; Zhu Z.; Huang L.; Cheng C. High Sensitivity UV Photodetectors Based on Low-Cost TiO 2 P25-Graphene Hybrids. Nanotechnology 2022, 33 (8), 08LT01 10.1088/1361-6528/ac3a37. 34787105

[ref34] Yu Y.; Wen W.; Qian X. Y.; Liu J. B.; Wu J. M. UV and Visible Light Photocatalytic Activity of Au/TiO 2 Nanoforests with Anatase/Rutile Phase Junctions and Controlled Au Locations. Sci. Rep. 2017, 7, 41253 10.1038/srep41253. 28117448 PMC5259751

[ref35] Luna M.; Barawi M.; Gómez-Moñivas S.; Colchero J.; Rodríguez-Peña M.; Yang S.; Zhao X.; Lu Y.-H.; Chintala R.; Reñones P.; et al. Photoinduced Charge Transfer and Trapping on Single Gold Metal Nanoparticles on TiO 2. ACS Appl. Mater. Interfaces 2021, 13 (42), 50531–50538. 10.1021/acsami.1c13662. 34641675 PMC8554764

[ref36] Arshad M. S.; Trafela Š.; Rožman K. Ž.; Kovač J.; Djinović P.; Pintar A. Determination of Schottky Barrier Height and Enhanced Photoelectron Generation in Novel Plasmonic Immobilized Multisegmented (Au/TiO 2) Nanorod Arrays (NRAs) Suitable for Solar Energy Conversion Applications. J. Mater. Chem. C 2017, 5 (40), 10509–10516. 10.1039/C7TC02633A.

[ref37] Fowler R. H. The Analysis of Photoelectric Sensitivity Curves for Clean Metals at Various Temperatures. Phys. Rev. 1931, 38 (1), 45–56. 10.1103/PhysRev.38.45.

[ref38] Ouyang W.; Teng F.; Jiang M.; Fang X. ZnO Film UV Photodetector with Enhanced Performance: Heterojunction with CdMoO 4 Microplates and the Hot Electron Injection Effect of Au Nanoparticles. Small 2017, 13 (39), 1702177 10.1002/smll.201702177. 28834210

[ref39] Lee H.; Song K.; Lee M.; Park J. Y. In Situ Visualization of Localized Surface Plasmon Resonance-Driven Hot Hole Flux. Adv. Sci. 2020, 7 (20), 2001148 10.1002/advs.202001148. PMC7578898 33101854

[ref40] Yan T.; Cai S.; Hu Z.; Li Z.; Fang X. Ultrafast Speed, Dark Current Suppression, and Self-Powered Enhancement in TiO 2 -Based Ultraviolet Photodetectors by Organic Layers and Ag Nanowires Regulation. J. Phys. Chem. Lett. 2021, 12 (40), 9912–9918. 10.1021/acs.jpclett.1c03090. 34612650

[ref41] Wu K.; Chen J.; McBride J. R.; Lian T. Efficient Hot-Electron Transfer by a Plasmon-Induced Interfacial Charge-Transfer Transition. Science 2015, 349 (6248), 632–635. 10.1126/science.aac5443. 26250682

[ref42] Giugni A.; Torre B.; Toma A.; Francardi M.; Malerba M.; Alabastri A.; Zaccaria R. P.; Stockman M. I.; Di Fabrizio E. Hot-Electron Nanoscopy Using Adiabatic Compression of Surface Plasmons. Nat. Nanotechnol. 2013, 8 (11), 845–852. 10.1038/nnano.2013.207. 24141538

[ref43] Zhang J.; Toe C. Y.; Kumar P.; Scott J.; Amal R. Engineering Defects in TiO2 for the Simultaneous Production of Hydrogen and Organic Products. Appl. Catal., B 2023, 333, 122765 10.1016/j.apcatb.2023.122765.

[ref44] Nishijima Y.; Ueno K.; Yokota Y.; Murakoshi K.; Misawa H. Plasmon-Assisted Photocurrent Generation from Visible to near-Infrared Wavelength Using a Au-Nanorods/TiO2 Electrode. J. Phys. Chem. Lett. 2010, 1 (13), 2031–2036. 10.1021/jz1006675.

[ref45] Shi X.; Kosei U.; Naoki T.; Hiroaki M. Plasmon-Enhanced Photocurrent Generation and Water Oxidation with a Gold Nanoisland-Loaded Titanium Dioxide Photoelectrode. J. Phys. Chem. C 2013, 117, 2494–2499. 10.1021/jp3064036.

[ref46] Kumbhar S. M.; Shevate S. S.; Patil A. R.; Shaikh S. K.; Rajpure K. Y. Dip Coated TiO2 Based Metal-Semiconductor-Metal Ultraviolet Photodetector for UV A Monitoring. Superlattices Microstruct. 2020, 141, 106490 10.1016/j.spmi.2020.106490.

[ref47] Joshna P.; Hazra A.; Chappanda K. N.; Pattnaik P. K.; Kundu S. Fast Response of UV Photodetector Based on Ag Nanoparticles Embedded Uniform TiO 2 Nanotubes Array. Semicond. Sci. Technol. 2020, 35 (1), 015001 10.1088/1361-6641/ab52f1.

[ref48] Gao X. D.; Fei G. T.; Xu S. H.; Zhong B. N.; Ouyang H. M.; Li X. H.; Zhang L. De. Porous Ag/TiO2-Schottky-Diode Based Plasmonic Hot-Electron Photodetector with High Detectivity and Fast Response. Nanophotonics 2019, 8 (7), 1247–1254. 10.1515/nanoph-2019-0094.

[ref49] Ghosh C.; Dwivedi S. M. M. D.; Ghosh A.; Dalal A.; Mondal A. A Novel Ag Nanoparticles/TiO2 Nanowires-Based Photodetector and Glucose Concentration Detection. Appl. Phys. A 2019, 125 (12), 810 10.1007/s00339-019-3108-5.

[ref50] Xie Y.; Wei L.; Li Q.; Wei G.; Wang D.; Chen Y.; Jiao J.; Yan S.; Liu G.; Mei L. Self-Powered Solid-State Photodetector Based on TiO2 Nanorod/Spiro-MeOTAD Heterojunction. Appl. Phys. Lett. 2013, 103 (26), 261109 10.1063/1.4858390.

[ref51] Das Mahapatra A.; Das A.; Ghosh S.; Basak D. Defect-Assisted Broad-Band Photosensitivity with High Responsivity in Au/Self-Seeded TiO 2 NR/Au-Based Back-to-Back Schottky Junctions. ACS Omega 2019, 4 (1), 1364–1374. 10.1021/acsomega.8b03084. 31459404 PMC6648538

[ref52] Pescaglini A.; Martín A.; Cammi D.; Juska G.; Ronning C.; Pelucchi E.; Iacopino D. Hot-Electron Injection in Au Nanorod–ZnO Nanowire Hybrid Device for Near-Infrared Photodetection. Nano Lett. 2014, 14 (11), 6202–6209. 10.1021/nl5024854. 25313827

[ref53] Wang W.; Klots A.; Prasai D.; Yang Y.; Bolotin K. I.; Valentine J. Hot Electron-Based Near-Infrared Photodetection Using Bilayer MoS 2anmu Yang, Kirill I. Bolotin, and Jason Valentine. Nano Lett. 2015, 15 (11), 7440–7444. 10.1021/acs.nanolett.5b02866. 26426510

[ref57] Sessolo M.; Bolink H. J. Hybrid Organic-Inorganic Light-Emitting Diodes. Adv. Mater. 2011, 23 (16), 1829–1845. 10.1002/adma.201004324. 21344510

[ref58] Jin Y.; Wang J.; Sun B.; Blakesley J. C.; Greenham N. C. Solution-Processed Ultraviolet Photodetectors Based on Colloidal ZnO Nanoparticles. Nano Lett. 2008, 8 (6), 1649–1653. 10.1021/nl0803702. 18459745

[ref59] Ibrahem M. A.; Rasheed B. G.; Mahdi R. I.; Khazal T. M.; Omar M. M.; O’Neill M. Plasmonic-Enhanced Photocatalysis Reactions Using Gold Nanostructured Films. RSC Adv. 2020, 10 (38), 22324–22330. 10.1039/D0RA03858J. 35514594 PMC9054582

[ref60] Li J.; Cushing S. K.; Meng F.; Senty T. R.; Bristow A. D.; Wu N. Plasmon-Induced Resonance Energy Transfer for Solar Energy Conversion. Nat. Photonics 2015, 9 (9), 601–607. 10.1038/nphoton.2015.142.

[ref62] Georgakopoulos T.; Todorova N.; Karapati S.; Pomoni K.; Trapalis C. Photoconductivity Studies on Surface Modified TiO2 Nanoparticles. Mater. Sci. Semicond. Process. 2019, 99, 175–181. 10.1016/j.mssp.2019.04.027.

[ref63] Majumder S.; Jana S. K.; Bagani K.; Satpati B.; Kumar S.; Banerjee S. Fluorescence Resonance Energy Transfer and Surface Plasmon Resonance Induced Enhanced Photoluminescence and Photoconductivity Property of Au–TiO2Metal–Semiconductor Nanocomposite. Opt. Mater. 2015, 40, 97–101. 10.1016/j.optmat.2014.12.001.

[ref64] Wang W.; Zhang C.; Qiu K.; Li G.; Zhai A.; Hao Y.; Li X.; Cui Y. Enhancing Hot-Electron Photodetection of a TiO2/Au Schottky Junction by Employing a Hybrid Plasmonic Nanostructure. Materials 2022, 15 (8), 2737 10.3390/ma15082737. 35454430 PMC9025816

[ref65] Noothongkaew S.; Han J. K.; Lee Y. B.; Thumthan O.; An K.-S. Au NPs Decorated TiO 2 Nanotubes Array Candidate for UV Photodetectors. Prog. Nat. Sci.: Mater. Int. 2017, 27 (6), 641–646. 10.1016/j.pnsc.2017.10.001.

[ref66] Zhang Y.; Zhou R.; Xu R.; Fang L.; Zhou J.; Chen Y.; Ruan S. Visible-Blind Self-Powered Ultraviolet Photodetector Based on CuI/TiO 2 Nanostructured Heterojunctions. ACS Appl. Nano Mater. 2022, 5 (11), 16804–16811. 10.1021/acsanm.2c03776.

[ref70] Schneider J.; Matsuoka M.; Takeuchi M.; Zhang J.; Horiuchi Y.; Anpo M.; Bahnemann D. W. Understanding TiO2photocatalysis: Mechanisms and Materials. Chem. Rev. 2014, 114 (19), 9919–9986. 10.1021/cr5001892. 25234429

[ref71] Zhang X.; Chen Y. L.; Liu R. S.; Tsai D. P. Plasmonic Photocatalysi. Rep. Prog. Phys. 2013, 76 (4), 046401 10.1088/0034-4885/76/4/046401. 23455654

