# Supplementary material for: Plasmons Enhancing Sub-Bandgap Photoconductivity in TiO2 Nanoparticles Film
Source: ACS Omega. 2024 Feb 20;9(9):10169–76. doi: 10.1021/acsomega.3c06932 (PMC10918839; doi:10.1021/acsomega.3c06932)
Supplement: Supplementary file 1 — ao3c06932_si_001.pdf [file ao3c06932_si_001.pdf]

Supporting Information

Plasmons Enhancing Sub-Bandgap Photoconductivity in TiO_2 Nanoparticles Film

Mohammed A. Ibrahem[1,2*], Emanuele Verrelli[3], Ali M. Adawi[3*], Jean-Sebastien G. Bouillard[3*] and Mary O'Neill[4*]

[1] Laser Science and Technology Branch, Applied Sciences Department, University of Technology-Iraq.

[2] UNAM-Institute of Materials Science and Nanotechnology and National Nanotechnology Research Center, Bilkent University, Ankara 06800, Turkey.

[3] Department of Physics and Mathematics, University of Hull, Cottingham Road, Kingston upon Hull HU6 7RX, United Kingdom.

[4] School of Science and Technology, Nottingham Trent University, Clifton Lane, Nottingham NG11 8NS, United Kingdom.

mohammed.a.ibrahem@uotechnology.edu.iq, a.adawi@hull.ac.uk, j.bouillard@hull.ac.uk, and mary.oneill@ntu.ac.uk

Keywords: Defects photoconductivity, Au nanoparticles, surface plasmon, resonant energy transfer, hot electrons

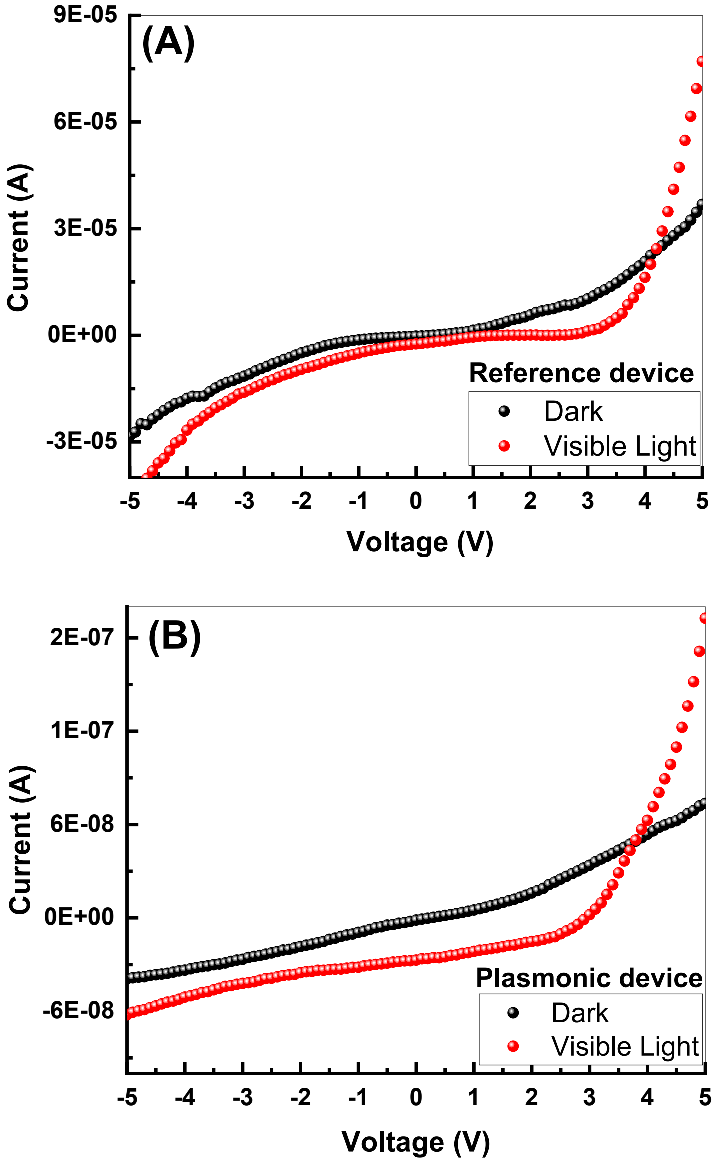

Figure S1: Shows the I-V curves under dark and visible irradiation at 546 nm of (A) reference device without Au nanoparticles. (B) the plasmonic device with Au nanoparticles.

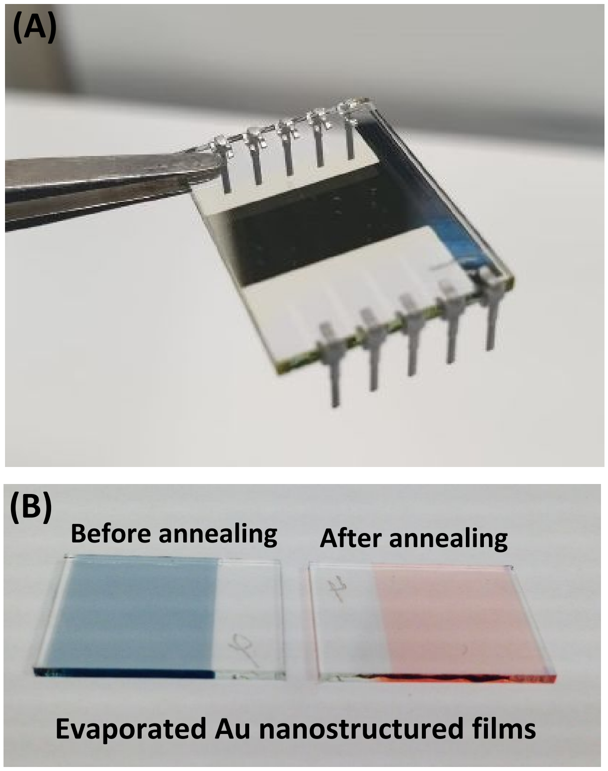

Figure S2: Shows (A) The photodetector device used in this work. The connection pins are used to switch between the photodetector devices and also help connecting the anode and the cathode with the measuring electrical circuit. (B) Au nanostructures film of 2 nm thick evaporated on a glass substrate before and after thermal annealing at 350 °C for 1 hour in air.

Fowler function:

The efficiency of hot electron injection at a given frequency is given by the Fowler equation [1]

$$F(\omega) = \frac{(\hbar\omega - E_B)^2}{4E_F\hbar\omega}$$

where ω is the angular frequency, E_F is the Fermi energy of the metal (here $E_F = 5.1\ eV$ for gold), and E_B is the energy barrier height at the interface (here $E_B = 1.7\ eV$).

References

(1) Fowler, R. H. The Analysis of Photoelectric Sensitivity Curves for Clean Metals at Various Temperatures. *Phys. Rev.* **1931**, *38* (1), 45–56. https://doi.org/10.1103/PhysRev.38.45.
